# From Molecule to Patient: A Biotech Perspective

**DOI:** 10.1002/cpt.1676

**Published:** 2019-11-20

**Authors:** Robert Langer, Ana Jaklenec, Giovanni Traverso, Daniel Hartman

**Affiliations:** ^1^ Koch Institute for Integrative Cancer Research Massachusetts Institute of Technology Cambridge Massachusetts USA; ^2^ Brigham and Women's Hospital Harvard Medical School Boston Massachusetts USA; ^3^ Department of Mechanical Engineering Massachusetts Institute of Technology Cambridge Massachusetts USA; ^4^ Global Health Division Bill and Melinda Gates Foundation Seattle Washington USA

**Drug delivery systems have had an enormous impact on human health and have potential for a profound impact in low‐income countries. Here, we discuss four examples of our research, including their potential for medical treatments in the developing world.**

## Controlled release

Vaccines have changed the course of infectious diseases and are largely responsible for the dramatic reduction in under 5‐year‐old mortality in the developing world. However, patients often do not return for second or subsequent injections. In 1979, we published the first single‐step method of vaccination.^1^ The Bill and Melinda Gates Foundation was interested in the feasibility of a single‐step comprehensive vaccination by creating microspheres that release their contents in distinct, delayed bursts without prior leakage.

**Figure 1:**
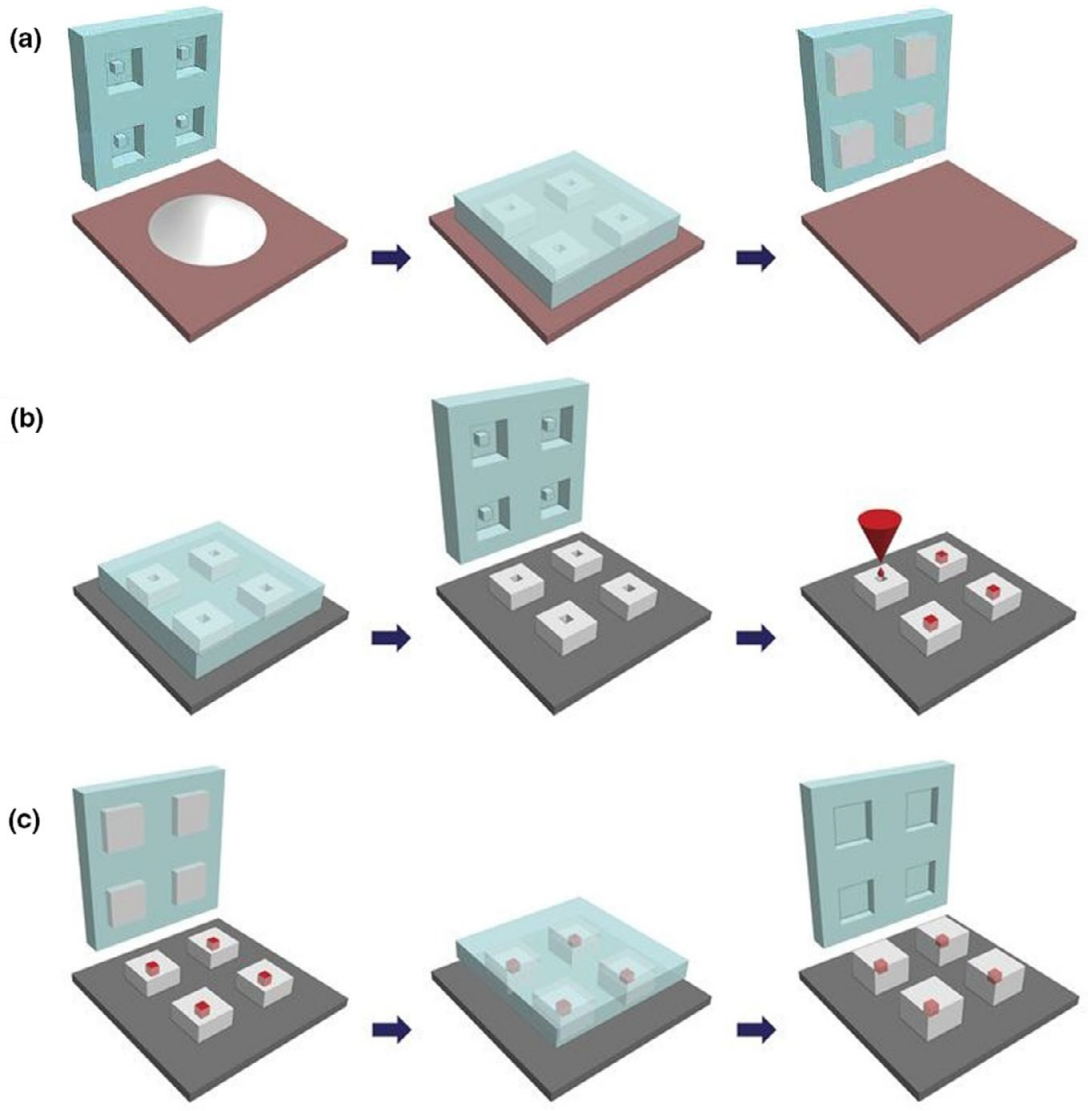
StampEd Assembly of polymer Layers‐fabricated controlled‐release microparticles. Particles are fabricated by (**a**) heating and pressing polymer between a patterned PDMS base mold and a Teflon surface, (**b**) transferring these bases to a new substrate and filling them with a model drug of interest, then (**c**) aligning an array of particle caps with drug‐filled bases and briefly applying a low amount of heat to sinter the two layers. (Adapted from McHugh *et al.* 2)

We thought of using polymers, in particular US Food and Drug Administration (FDA) approved polylactic/glycolic acid (PLGA), to accomplish this. First, we considered 3D printing of these microspheres. However, existing methods were not compatible with many FDA‐approved polymers or could damage the vaccine. Thus, we developed a new high‐resolution microstructure formulation approach called StampEd Assembly of polymer Layers, which combines technology used for computer chip manufacturing with soft lithography and sintering processes to produce small polymeric structures (**Figure** 1). We created a number of different PLGA microparticles, each intended to deliver at a different predetermined, desired time period, pulses of antigens, creating a single injection vaccine irrespective of the regimen.^2^ For example, we fabricated microparticles using different PLGA polymers (by changing molecular weight or LA/GA ratio, or both) with varying properties and filled the microparticles with fluorescently labeled dextran. Particles composed of different PLGA compositions were released *in vitro* at 10, 15, 34, 98, 126, 180 days, and nearly 300 days, respectively. No leakage was observed prior to release, indicating that this system releases its contents as a sharp pulse after degradation of the polymer barrier. When the particles were injected into mice, release times were almost identical to those *in vitro*. Particles could also be lyophilized or frozen at −20°C without altering release kinetics.^2^ We are now exploring these systems for the delivery of vaccines to prevent polio and other diseases.

Another problem is that patients often do not take their medicine, costing up to US $289 billion a year and hundreds of thousands of deaths in the United States alone^3^; this problem is worse in the developing world where availability and transportation issues are significant hurdles to access. To address this problem, we developed very‐long‐acting oral dosage formulations (1 week or more) that could, in some cases, last for the entire course of treatment to improve patient compliance. We hypothesized that for an oral sustained delivery dosage form to have a very prolonged gastric residence, it should: (i) have a shape and size (such as a capsule, or be able to be placed in a capsule) that can be ingested; (ii) have the ability to adopt a conformation that enables it to be placed in a capsule (or similar swallowable structure), but when it enters the gastric cavity and the capsule dissolves, it adopts a second shape that is so large that it prevents passage through the pylorus, but still allows food to pass through it; (iii) be able to carry substantial drug loadings; (iv) provide controlled release (for weeks or for whatever time period desired); (v) maintain drug stability in a low‐pH gastric environment; and (vi) degrade or dissociate into forms that can exit the stomach and pass through the gastrointestinal lumen with no potential for obstruction or perforation.^4^

**Figure 2:**
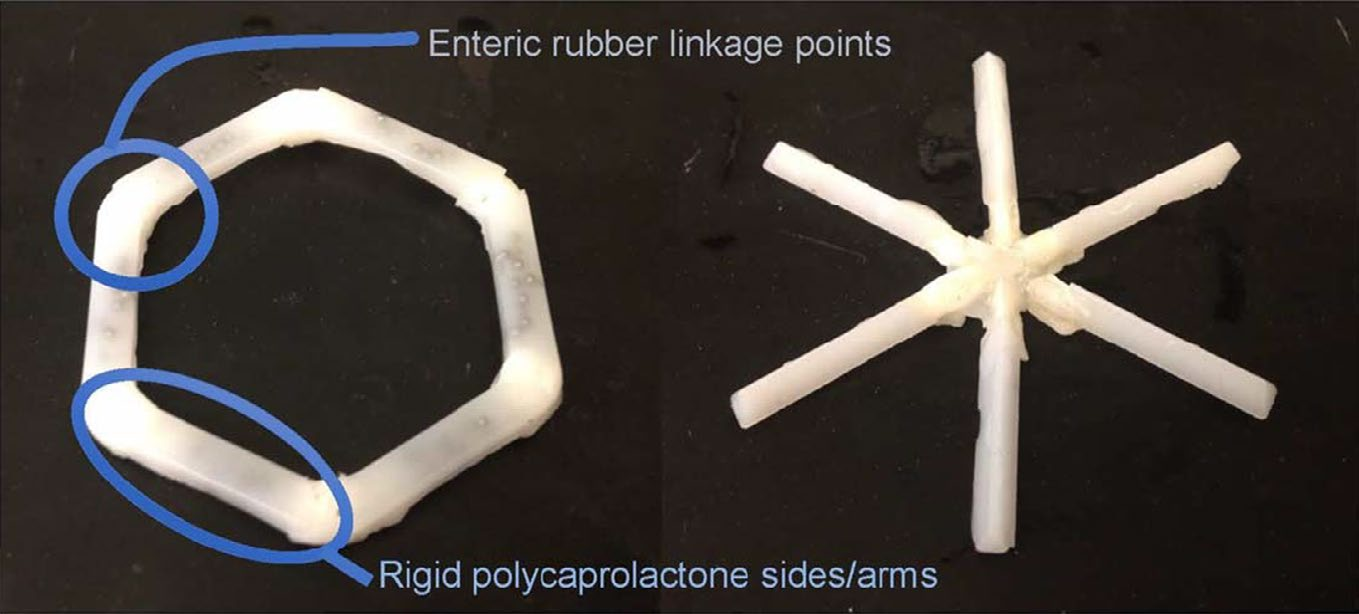
Initial prototypes. Design of a gastric residence vehicle. Two families of geometric arrangements of flexible and rigid elements able to fit into a capsule. (This figure is adapted from a figure that originally appeared in Bellinger *et al.* 4)

Several designs that fulfilled the above criteria (**Figure** 2), such as a “polygon” of alternating rigid and flexible elements, or a “stellate” or “star‐shaped” system. We synthesized such systems for a potential new treatment for malaria involving mass drug administration (MDA). The effectiveness of MDA depends on obtaining sufficient and prolonged drug blood levels in the majority of the population, which can be difficult in resource‐constrained or remote locations. Nonadherence, a well‐recognized barrier to effective care in the developed world, has also been shown to contribute to MDA failure.^4^

Ivermectin is a safe drug that is active against lymphatic filariasis, and other neglected tropical diseases, such as onchocerciasis and head lice, which infects millions of people worldwide, and is effective for the control of scabies during MDA campaigns. In addition, ivermectin kills the mosquito that transmits malaria. Oral ivermectin, with a half‐life of 18 hours in humans, achieves serum concentrations that kill the mosquito after a blood meal and prevents malaria transmission to another person. Serum levels of 8 ng/mL are sufficient to achieve this effect. This strategy has the potential to effectively interrupt vector transmission of malaria and could reduce prevalence within endemic regions. We developed a single‐encounter oral, ultra‐long‐acting formulation of ivermectin that achieved sustained therapeutic serum drug concentrations in porcine models above 8 ng/mL for > 2 weeks or more.^4^ We have also used these systems to release three different drugs for treatment and prevention of HIV.^5^ These very‐long‐acting delivery systems have now been tested with model drugs in humans.

## Lung delivery

Drug delivery systems to the lung often incorporate chlorofluorocarbon propellants, which may be environmentally dangerous, and many systems do not deliver the drug reproducibly or efficiently; generally, < 10% of the drug is delivered to the lung from the device due, in part, to aerosol aggregation because the aerosols are small (about 2 μm diameter). In addition, repeated delivery every few hours is often necessary.

Scientists classically attempted to address this issue by designing inhalers that could break apart aerosol aggregates or have other features. We took a radically different approach—designing new aerosols by changing aerosol geometry. Although prior work involved using small diameter (about 2 mm diameter) nonporous aerosols, we created large (5–20 µm), highly porous aerosols with extremely low densities. By lowering their density, we hypothesized that aerosol aerodynamics would be changed making it possible for unusually large particles to enter the lungs through an airstream. We further hypothesized that increasing aerosol particle size would lead to decreased particle aggregation, creating greater inhalation efficiency, as well as decreased phagocytosis by lung macrophages. The decreased phagocytosis can result in sustained drug release. We created such aerosols (**Figure** **S1**) and found that over 10 times the number of molecules could be delivered this way, compared with conventional aerosols and inhalers. The molecules could also be delivered to animals using these particles for over 4 days from a single dose.^6^ This capability enables simple small inhalers that can contain 70 mg of substance (before this, the delivery of 10 mg was generally not possible) to be delivered in a single dose. These aerosols have led to new treatments (Inbrija*)* for Parkinson's disease and other diseases. We are working with the Gates Foundation to develop systems for treating respiratory diseases (e.g., delivering lung surfactant) in the developing world.

## Microchip delivery

For many years, a problem central to the field of controlled‐release technology is that all systems displayed drug release rates that were either constant or decayed with time. There had been no way to change or modulate the release rate on demand, once release has commenced. We have examined magnetic, ultrasound, and electrical approaches that address this issue.

In the latter case, we developed a solid‐state silicon microchip that can provide controlled release of single or multiple chemical substances on demand.^7^ The release mechanism is based on the electrochemical dissolution of thin anode membranes, covering micro‐reservoirs filled with active agents.

This microchip has no moving parts. Release from a particular reservoir is initiated by applying an electric potential between the anode membrane covering that reservoir and a cathode. **Figure** **S2** shows a cut‐away portion of a prototype microchip containing reservoirs filled with the agent to be released. To cover the reservoirs, gold was originally chosen as a model membrane material because it is easily deposited and patterned, and resists corrosion in many solutions. However, the presence of a small amount of chloride ion creates an electric potential region, which favors the formation of soluble gold chloride complexes. Other metals, such as copper or titanium, tend to dissolve spontaneously under these conditions or do not form soluble materials on application of an electric potential. Although it is used as a model compound, gold has also been shown to be biocompatible.^7^ Subsequently, we also found platinum alloys to be useful.^8^

Our initial experiments showed that pulsatile release of a single compound or multiple compounds could be obtained.^7^ These chips are now being studied in humans. The first clinical trial of an implantable microchip‐based drug delivery device involved a human parathyroid hormone fragment (hPTH(1‐34)) being delivered from the device to treat osteoporosis, which normally requires daily injections, making patient compliance an issue (only 23% of patients maintain treatment). Furthermore, an increase in bone mineral density requires pulsatile hPTH(1‐34) delivery, which most implantable drug delivery products cannot achieve. The microchip‐based devices, containing discrete doses of lyophilized hPTH(1‐34), were implanted in 8 osteoporotic postmenopausal women for 4 months and wirelessly programmed to release doses from the device. A computer‐based programmer, operating in the Medical Implant Communications Service band, established a bidirectional wireless communication link with the implant to program the dosing schedule and receive implant status confirming proper operation.^8^

The electrically controlled release device dosing produced similar pharmacokinetics to multiple injections and had lower coefficients of variation. Bone marker evaluation indicated that daily release from the device increased bone formation. There were no adverse events and no quality‐of‐life changes.^8^ We are now also working with the Bill and Melinda Gates Foundation to create a very‐long‐acting (e.g., 10 years) microchip birth control system that women can turn on and off when desired to aid in family planning.

## Funding

This work is supported by the Bill and Melinda Gates Foundation and the National Institutes of Health.

## Conflict of Interest

R.L. discloses potential competing interests due to his affiliation with Microchips Biotech, Lyndra. For a list of entities with which R.L. is involved, compensated, or uncompensated, see http://www.dropbox.com/s/yc3xqb5s8s94v7x/Rev%2520Langer%2520COI.pdf?dl=0. Complete details of all relationships for profit and not for profit for G.T. can found at the following link: https://www.dropbox.com/sh/szi7vnr4a2ajb56/AABs5N5i0q9AfT1IqIJAE-T5a?dl=0. A.J. has no conflicts or competing interests to disclose. D.H. has no conflicts or competing interests to disclose.

## Supporting information


**Figure S1**. Adapted from Edwards, D., Hanes, J., Caponetti, G., Hrkach, J., Ben‐Jebria, A, Eskew, M., Mintzes, J. Deaver, D., Lotan, N., Langer, R. Large porous aerosols for pulmonary drug delivery. Science, 276: 1868–1871, 1997. Reprinted with permission from AAAS.

**Figure S2**. Adapted from Santini et al, 1999.
