# Supplementary figures and images for: From Molecule to Patient: A Biotech Perspective
Source: Clin Pharmacol Ther. 2019 Nov 20;107(1):65–7. doi: 10.1002/cpt.1676 (PMC7082779; doi:10.1002/cpt.1676)

**Figure S1**

Adapted from Edwards et al., Science 1997


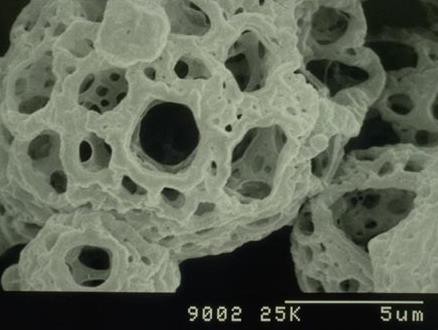

Supplement: Supplementary file 1 — Figure S1. Adapted from Edwards, D., Hanes, J., Caponetti, G., Hrkach, J., Ben‐Jebria, A, Eskew, M., Mintzes, J. Deaver, D., Lotan, N., Langer, R. Large porous aerosols for pulmonary drug delivery. Science, 276: 1868–1871, 1997. Reprinted with permission from AAAS. [file CPT-107-65-s001.docx]

# Figure S2


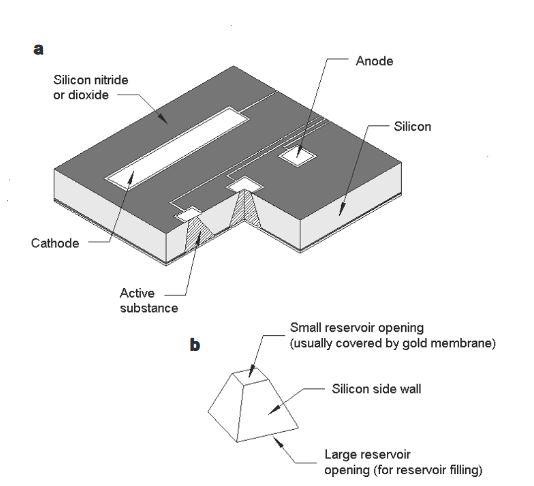


Adapted from Santini et al, 1999

Supplement: Supplementary file 2 — Figure S2. Adapted from Santini et al, 1999. [file CPT-107-65-s002.docx]
